# Complexity and phase transitions in citation networks: insights from artificial intelligence research

**DOI:** 10.3389/frma.2024.1456978

**Published:** 2024-09-25

**Authors:** Ariadne A. Costa, Rafael B. Frigori

**Affiliations:** ^1^Grupo de Redes Complexas Aplicadas de Jataí (GRAJ), Instituto de Ciências Exatas e Tecnológicas, Universidade Federal de Jataí (UFJ), Jataí, GO, Brazi; ^2^Universidade Tecnológica Federal do Paraná, Toledo, PR, Brazil

**Keywords:** citation network, artificial intelligence, Shannon entropy, fractal dimension, complex networks

## Abstract

In this study, we analyze the changes over time in the complexity and structure of words used in article titles and the connections between articles in citation networks, focusing on the topic of artificial intelligence (AI) up to 2020. By measuring unpredictability in word usage and changes in the connections between articles, we gain insights into shifts in research focus and diversity of themes. Our investigation reveals correspondence between fluctuations in word complexity and changes in the structure of citation networks, highlighting links between thematic evolution and network dynamics. This approach not only enhances our understanding of scientific progress but also may help in anticipating emerging fields and fostering innovation, providing a quantitative lens for studying scientific domains beyond AI.

## 1 Introduction

The study of citation networks is pivotal in understanding the progression and dissemination of scientific knowledge within various research communities. These networks are directed graphs, where each node represents an article, and each edge points from one article to another that it cites (or the opposite: to an article from another that cited it, depending on the implementation). They form a complex web of scholarly communication, reflecting how ideas propagate, gain attraction, and evolve over time (Newman, 2001).

Here we focus on a citation network of artificial intelligence (AI) papers, which is a branch of computer science that aims to create systems capable of performing tasks that would normally require human intelligence (Jiang et al., 2022). Understanding the dynamics of citation networks in AI research provides a unique lens through which we can observe the evolution of this rapidly advancing field. By examining the citation patterns, we can uncover how foundational ideas have emerged, transformed, and influenced subsequent work. This temporal analysis is crucial for identifying critical periods and key shifts in research focus, methodology, and application areas.

**Objective:** The primary objective of this study is to propose new methods for analyzing complex networks, particularly citation networks in AI research. The sub-objectives include:

- To track the evolution of the network's fractal dimension and the Shannon entropy of titles over time.
- To analyze the relationship between the diversity of research titles and the overall connectivity of the citation network.
- To provide a novel perspective on the dynamic and non-linear interactions in AI research and development.

### 1.1 Literature review

Different articles provided a comprehensive overview of the advancements and emerging themes in artificial intelligence (AI) research, utilizing various methodologies to track and analyze developments in the field. For instance, Liu et al. (2018) highlights the rapid growth and increasing collaboration in AI research, identifying key topics and influential entities over the first 16 years of the 21st century. On the other hand, Shao et al. (2021) explores the phenomenon of research convergence, where scholars from different regions show increasingly similar research interests, and applies principles like Pareto's rule to assess the distribution of scholarly output and influence in the AI domain.

Complementing these perspectives, Soliman et al. (2023) explore the major trends and emerging themes in AI research, particularly in the context of the COVID-19 era, highlighting how the pandemic has influenced research priorities and accelerated innovation in AI. Tang et al. (2020) focus on the pace of AI innovations, analyzing the factors that drive the speed of developments, including talent dynamics and the trial-and-error processes inherent in AI research.

Recent studies have further expanded on these analyses by applying bibliometric methods to trace the evolution of AI over the past decade, particularly focusing on the significant advancements in deep learning, self-learning algorithms, and reinforcement learning. These studies provide a comprehensive overview of the research landscape and forecast future trends in AI development (Shao et al., 2022). Additionally, the impact of AI on higher education has been explored through a combination of bibliometric analysis and topic modeling, which reveals the rapid growth in research output and the emergence of distinct thematic clusters in this domain (Maphosa and Maphosa, 2023). Moreover, a broad bibliometric analysis of AI research over the last 20 years offers insights into the evolution and current status of AI technologies, highlighting key trends and future directions (Gao and Ding, 2022). This analysis revealed a significant increase in publications in recent years, with China emerging as the largest contributor, reflecting the broad application of AI in information science (Hussain and Ahmad, 2024).

While these studies offer valuable insights into the development of AI, they primarily focus on the surface-level dynamics of research output and influence. However, they do not deeply explore the underlying structural complexities of citation networks and the phase transitions that may occur within these networks. Therefore, our primary objective is to propose new methods for analyzing complex networks, while also providing a novel perspective on the dynamic and non-linear interactions in AI research and development.

Since citation networks can be viewed as complex systems with self-similarities and scaling behavior related to fractal structures (Skums and Bunimovich, 2020), tracking the evolution of the network's fractal dimension (Wei et al., 2014) and the Shannon entropy of titles (Shannon, 1948) over time can enhance the analysis, providing a dynamic understanding of the network's development (Clough and Evans, 2016; Bentley and Maschner, 2000; Ramirez-Arellano et al., 2023). Temporal analysis of these metrics can reveal shifts in research paradigms, the rise of influential publications, and the diffusion of scientific innovations. Changes in the fractal dimension may indicate periods of rapid expansion or stabilization of research fields, while variations in Shannon entropy can highlight changes in the diversity and distribution of topics (Vale Cunha et al., 2020). This integrated approach offers a comprehensive view of the emergent properties and evolutionary dynamics of citation networks, deepening our understanding of scientific progress and information dissemination.

Simultaneously with the microscopic analyses, we computed the macroscopic properties of the citation network, specifically the average degree of the network for each year and its time derivative. The average degree indicates the connectivity and citation density within the network, providing insights into how interlinked the AI research community has become over time. By plotting the annual entropy alongside the derivative of the average degree, we investigate the relationship between the diversity of research themes (a microscopic property) and the overall change of connectivity of the citation network (a macroscopic property). Our findings suggest a notable similarity between these two measures, indicating that periods of high thematic diversity often coincide with increased scholarly connectivity. These changes are intriguingly related to the particular growth changes seen in the fractal dimension, indicating a “thermostatistical” connection relating complexity in such system.

## 2 Methods

### 2.1 Data acquisition and preprocessing

The citation network data was obtained from MAG (Microsoft Academic Graph), comprising information regarding paper citations. The raw data was provided by the Collaborative Archive & Data Research Environment (CADRE) project at Indiana University (Mabry et al., 2020). The specific data set used here builds upon source previously selected and published by Benatti et al. (2023). The authors filtered the data for publications of AI up to 25th June, 2020 (when MAG was then discontinued). The citation network was generated by selecting titles and abstracts containing at least one of the predefined keywords (complete list bellow), all citations between these selected documents were considered. Non-connected documents were excluded and only the largest connected component of the resulting network was kept. The resulting network has 897,991 nodes (articles), 10,713,033 edges (citations) and an average degree of 11.93 (citation per article).

- Linear discriminant analysis
- Maximum entropy classifier
- Decision list
- K-nearest-neighbor
- Neural network
- Support vector machine
- Categorical mixture model
- K-means clustering
- Kernel PCA
- Bootstrap aggregating
- Mixture of expert
- Markov random field
- Particle filter
- Kriging
- Independent component analysis
- Conditional random field
- Markov model
- Dynamic time warping
- Quadratic discriminant analysis
- Decision tree
- Kernel estimation
- Naive Bayes classifier
- Perceptron
- Gene expression programming
- Hierarchical clustering
- Correlation clustering
- Boosting
- Ensemble averaging
- Bayesian network
- Kalman filter
- Gaussian process regression
- Linear regression
- Principal component analysis
- Maximum entropy
- Hidden Markov model

### 2.2 Word cloud generation

We conducted a comprehensive analysis of research trends by examining the frequency of word appearances in the titles of papers within a citation network. Titles, despite their brevity, serve as effective proxies for full-text content, providing clear indicators of research themes and shifts in academic focus.The titles of papers were subjected to a rigorous text preprocessing pipeline to ensure the consistency and reliability of the analysis. In this process we employed the NLTK library (Bird et al., 2009), which included:

- **Punctuation and Special Character Removal**: All non-alphabetic characters were removed to eliminate noise and irrelevant information.
- **Case Normalization**: Titles were converted to lowercase to maintain consistency in word representation, reducing the potential for discrepancies caused by capitalization.
- **Tokenization**: Titles were split into individual words or tokens, which were then analyzed separately.
- **Stopword Removal**: Common stopwords, such as “the,” “and,” and other frequently occurring but semantically irrelevant words, were removed using both the standard NLTK stopword list and a custom list tailored to exclude non-thematic words specific to research titles (e.g., “use,” “study,” and “data”).
- **Lemmatization and Stemming**: Words were lemmatized and stemmed to normalize different forms of the same word, ensuring that variations like “run,” “running,” and “ran” were treated as a single term. This dual approach of stemming and lemmatization enhanced the consistency of word representation, allowing for a more accurate thematic analysis.

To identify underlying research themes within the citation network, we employed Latent Dirichlet Allocation (LDA), a widely recognized probabilistic model used for topic modeling in text analysis (Blei et al., 2001, 2003). LDA assumes that documents are mixtures of topics, where each topic is a distribution over words. By analyzing the co-occurrence of words across the dataset, LDA infers the hidden thematic structure of the text. We applied LDA to the preprocessed titles within 3-year intervals, covering the period from 1968 to 2020. The LDA model was implemented using the LatentDirichletAllocation function from the scikit-learn library (Pedregosa et al., 2011). The LDA model was trained on the vectorized title data, with each title represented as a sparse matrix of word counts. The model then generated a set of topics, each defined by a distribution of words, reflecting the dominant themes in the research corpus during each 3-year window. The following parameters were used:

- **Number of Topics (n_components)**: Set to 10, thus the model identifies ten distinct themes per triennium. This value was found as the best average value by a systematic scan evaluating *perplexity* and *coherence* scores (Newman et al., 2010) using gensim,sklearn libraries (Pedregosa et al., 2011; Řehůřek and Sojka, 2010), which leads to optimal thematic granularity and interpretability.
- **Maximum Document Frequency (max_df)**: Set to 0.90, excluding too common words appearing in more than 90% of the titles as thematically non-informative.
- **Minimum Document Frequency (min_df)**: Set to 2, ensuring that only words appearing in at least two documents were considered, thereby filtering out noise from very rare words.
- **Random State (random_state)**: Set to 42, providing reproducibility of results by ensuring consistent output across different runs of the model.

Finally, word clouds were generated to visually represent the most relevant words. For each triennial time interval, up to 2 words from each of the 10 themes detected by LDA contributed to create clouds using the WordCloud library (Mueller, 2020). A consistent color was assigned to each word across all word clouds, allowing for visual coherence and making it easier to track the presence and prominence of research trends over time.

### 2.3 Shannon entropy calculation

The Shannon entropy has been widely applied to quantify informational content and variability measures in different systems (Isik, 2010; Eskov et al., 2017; Zachary and Dobson, 2021), for instance, also as an indicator of dynamical stability (Eskov et al., 2017; Cincotta et al., 2021). Traditionally, the study of phase transitions assume thermodynamic equilibrium and employ the canonical ensemble, which ensures entropy concavity, being signaled by regions in the phase diagram where a system undergoes a significant change in its state or behavior (Sethna, 2021). However, for the class of systems in which long-range interactions are not negligible, assumptions over local equilibrium might not be allowed so requiring a microcanonical description (Gross, 2001). In the context of citation networks, we are dealing with this class of systems (Li et al., 2007; Hung and Wang, 2010); phase transitions can manifest as sudden (disruptive) shifts in research focus or the emergence of new influential fields. These structural transitions are often marked by metastabilities or “convex intruders” in the microcanonical entropy, as described by Gross (2001) as anomalies where the entropy function deviates from its typical concave shape and becomes convex. These convex regions indicate phase coexistence, such as in nuclear fragmentation (Chomaz, 2002; Ogul et al., 2005) or water ice-melting (Raḿırez et al., 2018), where the system can exhibit negative specific heat and other unusual thermodynamic properties. Therefore, in the context of citation networks, convex intruders are expected to happen in Shannon entropy during (structural) phase transitions indicating emergence or significant reorganization of scientific fields, as also observed in physical systems as proteins (Nakagawa and Peyrard, 2006; Frigori et al., 2013; Frigori, 2017; Trugilho and Rizzi, 2022) and magnetic spins (Barré et al., 2001; Alves and Frigori, 2016).

Shannon entropy was computed as a function of words appearing in titles for each year to quantify the diversity or randomness of the words used. The following formula was utilized (Shannon, 1948):


(1) $$\begin{array}{l} H\left(t\right)=-\overset{n}{\underset{i=1}{\sum }}p\left(x_{i}\right)\log_{2}\left(p\left(x_{i}\right)\right), \end{array}$$

where *n* is the number of different words (or tokens) in the titles and *p*(*x*_*i*_) is the probability of word *x*_*i*_ occurring in the titles per year. While small-sized, titles effectively capture the essence of the research and serve as excellent proxies for abstracts and even the full content of the articles.

The smoothed Shannon entropy (*H*_S_) was computed to obtain a more refined and continuous representation of entropy over time, thereby enabling the identification of trends and fluctuations in word diversity. To this end, the Savitzky-Golay filter Schafer (2011), a digital filter known for its ability to smooth data without significantly distorting the signal, was applied to the selected entropy values. We tried various window sizes and we choose a polynomial order of 3 to balance smoothness and fidelity to the original data. The Savitzky-Golay filter is a function of the Scipy library (Virtanen et al., 2020).

Then, the values of *H*_S_ were normalized to a range of [0, 1] using MinMaxScaler from the Scikit-Learn library. This step ensured proper visualization scales for the entropy and the derivative of the average degree. To identify regions of entropy instability denoting structural phase transitions, the second derivative (curvature) of the smoothed and normalized entropy curve was calculated. Regions where the second derivative was positive ($\frac{d^{2}H_{S}\left(t\right)}{dt^{2}}>0$) indicated convexity and potential instability, while regions where the second derivative was negative ($\frac{d^{2}H_{S}\left(t\right)}{dt^{2}}<0$) indicated concavity and stability (Gross, 2001).

### 2.4 Average degree and its derivative

The citation network data was represented as a directed graph with papers as nodes and citations as edges. For each node (paper) in the citation network, its degree was calculated, representing its number of cited papers. This procedure was done for papers of each year, so that the average degree per year was computed. Since temporal changes in this quantity might be a signal of change in research activity, and so in citation patterns, we used it as a surrogate “order parameter” in which peaks in its numeric time derivative (i.e., *D*_t_ < *Degree* >) indicates the eventual phase transitions also expressed by the entropy.

### 2.5 Fractal dimension computation

Fractal dimension quantifies the complexity of a network by describing how its structure changes with scale and how densely it fills the space. The box-counting method (Wei et al., 2014; Sun and Zhao, 2014), a popular technique for computing fractal dimension, involves overlaying the network with a grid of size ϵ and counting the number of boxes *N*(ϵ) that contain at least one node. By varying ϵ and plotting log(*N*(ϵ)) against log(1/ϵ), the fractal dimension (*D*_frac_) is estimated from the slope of the linear fit to this log-log plot:


(2) $$\begin{array}{l} D_{\text{frac}}=\underset{\epsilon \to 0}{\lim}\frac{\log N\left(\epsilon \right)}{\log\frac{1}{\epsilon }}. \end{array}$$

By systematically decreasing ϵ and counting the occupied boxes, meaningful insights into the network's topology and its hierarchical organization can be derived. As more papers are published and added to the network, the fractal dimension can change in several ways:

1. **Growth and density:** Initially, as new papers are added, the network grows, increasing the potential complexity. This can increase the fractal dimension if the network becomes more interconnected and densely packed.
2. **Scaling behavior:** The fractal dimension measures how the number of connections scales with the size of the network. As more papers are added, if the connections grow in a self-similar manner, the fractal dimension might stabilize.
3. **Connectivity patterns:** If new papers are highly connected to a few existing ones (preferential attachment), it might increase local density but not necessarily the overall fractal dimension.
4. **Structural changes:** Introducing new fields or interdisciplinary papers can change the network's structure, potentially affecting the fractal dimension by altering connectivity patterns.
5. **Saturation:** At some point, adding more papers might not significantly increase the fractal dimension if the network reaches a saturation point in terms of connectivity.

Therefore, this method effectively captures the scaling behavior of the network, where a higher fractal dimension indicates a more complex and densely interconnected structure. This contributes to our understanding of the dynamics of scientific knowledge and information dissemination. To analyze this, we track changes in fractal dimension over time as new papers are added, observing how these changes relate to the newly introduced citations.

## 3 Results

**Figure 1:**
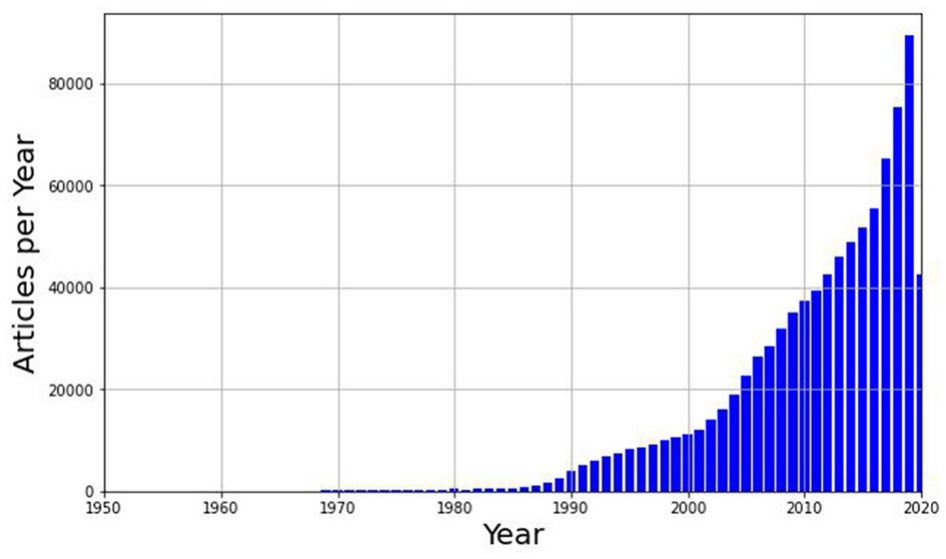
Histogram of publications per year, from 1950 to 2020, extracted from Microsoft Academic Graph (MAG).

To provide readers with a birds' eye graphic perspective of AI time evolution, we commence the analysis of the citation network by presenting a graphic showing the number of articles published annually from 1950 to 2020 (Figure 1). For some, the inception of artificial intelligence began with the publication of Turing's work in 1950 (Turing, 2009), while others consider the pivotal moment to be the conference held at Dartmouth in 1956. The scale of the figure does not allow for easy visualization, but there are indeed articles published between 1950 and 1970, starting from a single publication in 1950 and increasing to 159 in 1970. By observing the figure, we can identify a clearer picture of the development and increasing popularity of AI over the decades. It is worth mentioning that in 2020 the number of publications does not come close to that of the previous year as the data collected is only up to July 2020.Historically AI has transitioned from a niche academic field to having a direct impact on everyday life (Liu et al., 2018; Shao et al., 2021).

**Figure 2:**
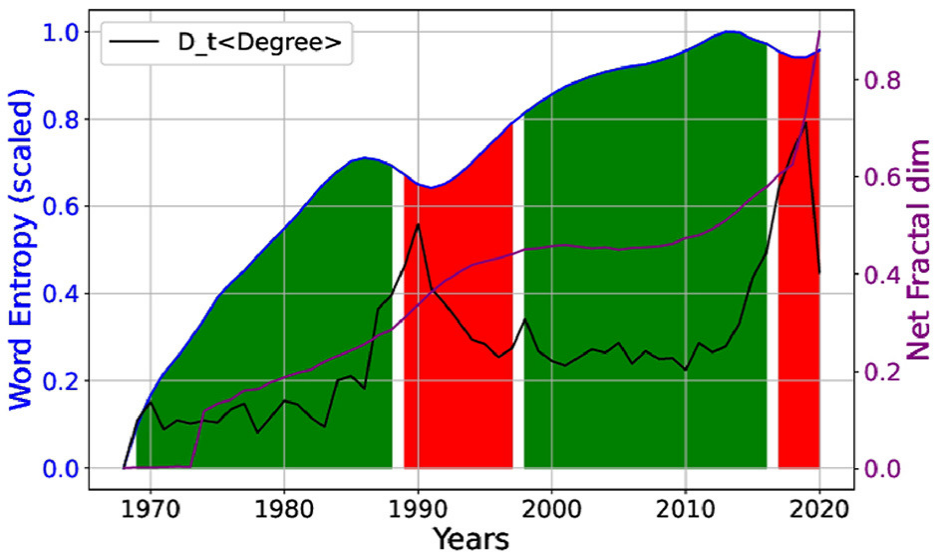
The scaled Shannon entropy as a function of word occurrence in paper titles along the years, metastabilities (red) correlate with peaks in the temporal derivative of normalized network average degree (*D*_t_ < *Degree* >) (black). The network fractal dimension (*D*_frac_) is displayed in (purple). The green regions presents stability.

It is interesting to consider the insights provided by information theory in understanding the trends within the field. Shannon entropy (Shannon, 1948), a cornerstone of information theory, measures the unpredictability or information content within a dataset. When applied to the occurrence of words in the titles of scientific papers, entropy can provide valuable information about the diversity and focus of research topics over time, particularly in AI. A high entropy indicates a wide range of topics and a diverse research landscape, whereas a low entropy suggests a concentration on specific topics. The examination of entropy metastability regions (Figure 2), identified by analyzing the second derivative of the entropy curve with respect to time, offered insights into temporal shifts in the diversity of words utilized in paper titles.

Noteworthy, two market metastabilities are observed in the entropy, coinciding with peaks in the derivative of the degree distribution (*D*_t_ < *Degree* >) around the 1990s and the end 2010s. These peaks indicate paradigm shifts in AI research due to the emergence of new methodologies. The observed peaks in (*D*_t_ < *Degree* >) simultaneously correlated with curvature changes in entropy are fingerprints, in the statistical mechanics language, of critical phase transitions of knowledge (i.e., revolutions) in AI. These transitions likely are driven by technological innovation and intellectual contributions.

These findings are in agreement with the monotonic increase in the network fractional dimension (*D*_*frac*_), which is not only an extensive function of the field growth shown by the increasing number of published papers, but also tracks its internal complexity change embedded on citation patterns as described in the methodology section. Deserves to be noted that by inspecting *D*_*frac*_(*t*) we can testify the life-cycle from the inception of a still incipient research area (*t*≈1970*s, the average D*_*frac*_ (< *D*_*frac*_>)≈0.15), clearly into a scenario we previously named “Growth and Density” and “Structural Changes”. This field grows and develops until it becomes an emerging field (t ≈ 1990s, < *D*_*frac*_>≈0.45) and then matures, within an intermediate period exhibiting “Scaling Behavior,” “Connectivity Patterns,” and “Saturation” whereas *D*_*frac*_(2000 < *t* < 2010) stays almost constant. Finally, a new phase emerges with a surge in complexity observed in the citation patterns, with higher interconnection and scale-invariance as time evolves after 2010s. This result shows an explosive growth of *D*_*frac*_ occurring almost at the beginning of the 2020s, likely feed not only by the exponential increase in published articles but also by their citation patterns.

**Figure 3:**
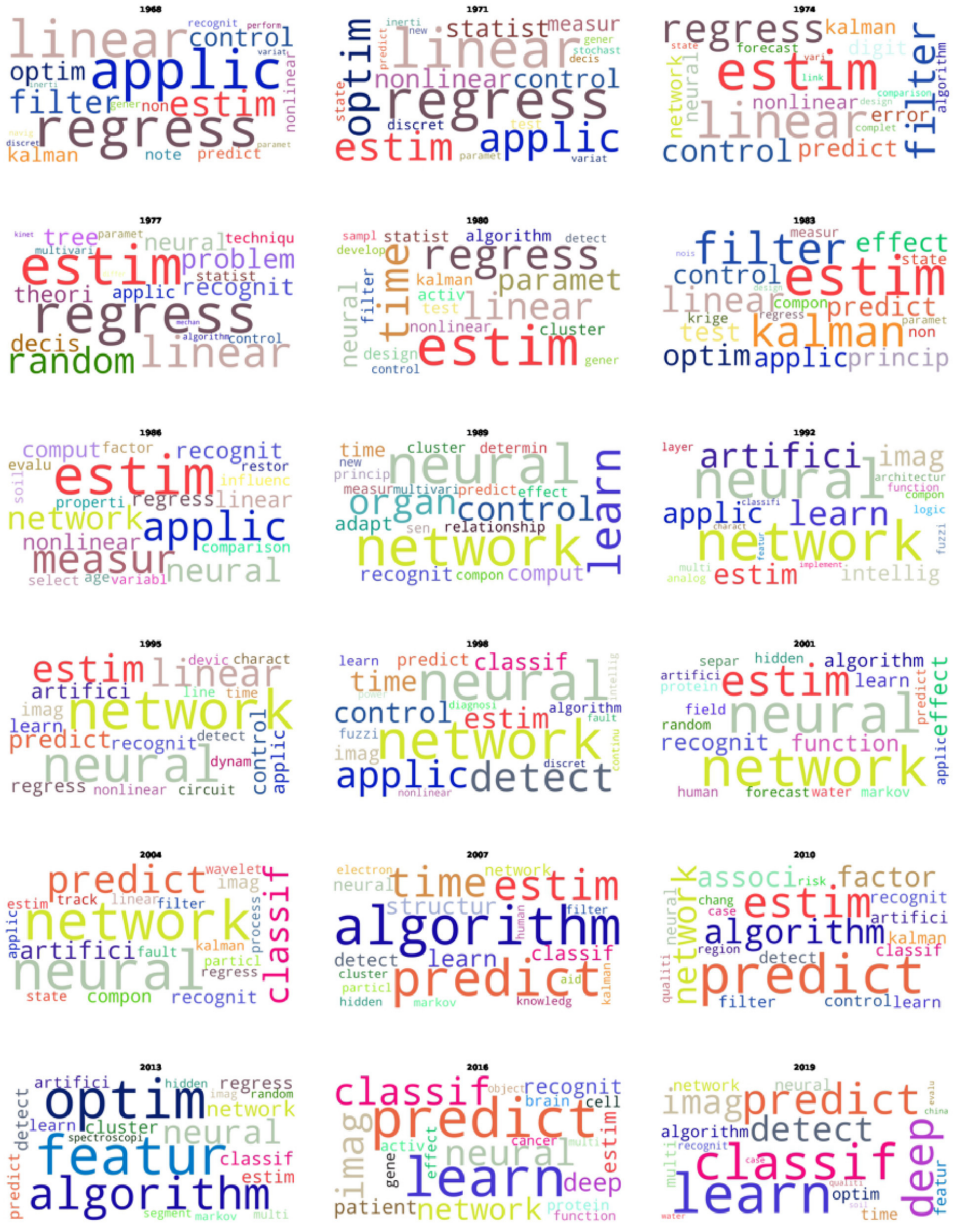
Word clouds showcasing up to the 20 most relevant words from 10 themes in the titles of AI articles published in triennial intervals between 1968 and 2019.

The observation of disruptive behaviors in citation networks within the field of artificial intelligence (AI) during the late 1980s to 1990s and again from the late 2010s to 2020 indicates significant paradigm shifts and increasing complexity. These shifts can be attributed to the emergence of new technologies, applications, and paradigms in AI. Figure 3 presents an overview of AI research by illustrating the evolution of the most frequently occurring words in the titles of publications from 1968 to 2019.

Following we intend to present possible interpretations of these shifts seen in Figure 2 based on historic events, which are also illustrated in our word clouds of Figure 3. In the late 1980s to 1990s, several key developments catalyzed a paradigm shift in AI [the first peak in the *D*_t_ < *Degree* > and instability in the scaled Shannon entropy accompanied by growth in *D*_frac_(t) exhibited in Figure 2]. Figure 3 shows the term “neural” emerging around 1986, gaining prevalence, and consistently maintaining its prominence thereafter. The period witnessed a resurgence in neural networks, specifically with the development of backpropagation algorithms for training multi-layer perceptrons. A publication by Rumelhart et al. (1986) was a critical moment, demonstrating that neural networks could learn data representations, thereby reinvigorating interest and research in this area. Concurrently, expert systems (Kastner and Hong, 1984), which had dominated the AI landscape in the early 1980s, began to show limitations. The complexity and brittleness of rule-based systems underscored the need for more flexible learning approaches, driving interest toward neural networks and other machine learning techniques. As observed from the word clouds, as these techniques emerged and gained strength, the use of the Kalman filter (Welch, 2021) and regression models (Fahrmeir et al., 2013) gradually lost their prominence, at the same epoch that *D*_frac_(t) remained stabilized.

The period from the late 2010s to 2020 witnessed another significant phase transition in AI (the second peak in *D*_t_ < *Degree* > and another instability appeared in the scaled entropy, which was concurrent to an explosive increase in *D*_frac_(t), as shown in Figure 2) ) marked by the deep learning (Bengio et al., 2013) revolution and advances in AI applications. In Figure 3, we observe that the words “deep” and “learn” appeared in small size in 2016 (indicating low occurrence), and subsequently increased in size by 2019, reflecting a growing usage in deep learning techniques. The success of AlexNet (Krizhevsky et al., 2012) in the ImageNet competition in 2012 (https://www.image-net.org/challenges/LSVRC/) demonstrated the potential of deep learning for image recognition tasks, leading to its widespread adoption across various domains.

Besides that, generative models and transfer learning techniques, such as generative adversarial networks (GANs) (Goodfellow et al., 2014) and variational autoencoders (VAEs) (Kingma and Welling, 2013), opened new possibilities for data generation and unsupervised learning. Transfer learning, exemplified by models like BERT (Devlin et al., 2018) and GPT (Radford et al., 2018), allowed for the transfer of knowledge from large pre-trained models to specific tasks, enhancing performance and reducing the need for large labeled datasets. Advances in natural language processing (NLP) were significant during this period, with models like Transformer architectures revolutionizing the field. Applications such as machine translation, sentiment analysis, and conversational agents saw substantial improvements, contributing to the observed paradigm shift. The continuous evolution of AI, driven by new methods, increased computational power, and broader application domains, underscores the dynamic nature of the field.

As we can see, the words observed in the word clouds are essentially related to the methodologies used rather than the problems to which they were applied. Interestingly, some of the terms identified as most prevalent over time by Shao et al. (2021) are absent from the word clouds generated in our analysis. Notably, terms like “Computer vision” and “Genetic algorithm” do not appear. These discrepancies are likely due to differences in the datasets used, which is a common limitation in studies of this nature, along with the temporal boundaries of the collected data.

## 4 Conclusions

The exponential growth in article publications reflects the growing importance of AI in modern society. Our investigation into Shannon entropy and phase transitions within artificial intelligence (AI) citation networks has yielded profound insights into the dynamic evolution of scientific research. Through the analysis of word occurrences in paper titles, Shannon entropy has emerged as a robust metric, revealing significant temporal fluctuations that correspond to critical shifts in AI research focus, reminiscent of phase transitions observed in physical systems. These entropy shifts correlate closely with peaks in the derivative of the average degree of citation networks, indicating periods of heightened connectivity and structural transformation within the field.

Furthermore, our exploration of the fractal dimension (*D*_frac_) clarified the evolving complexity and hierarchical organization of AI citation networks. Initially characterized by a lower *D*_frac_, reflecting a nascent phase, AI research progresses through phases of expansion and maturation, marked by increasingly interconnected and scale-invariant structures. This evolutionary trajectory underscores the emergence of influential clusters and pivotal papers, shaping the dissemination and impact of scientific knowledge.

The present integrated approach, combining entropy analysis, fractal dimension computation, and a macroscopic network property examination, provides a comprehensive understanding of AI research dynamics. Moreover, our findings advance the methodological analysis of citation networks and offer valuable insights into the historical and structural development of AI research. By bridging information theory with network science, our study underscores the interdisciplinary nature of scientific inquiry and establishes a quantitative framework for anticipating future trends and fostering innovation not only in AI but also across diverse scientific domains.

## Data Availability

Publicly available datasets were analyzed in this study. This data can be found here: https://zenodo.org/records/5578567.
